# Cellular and Molecular Progression of Prostate Cancer: Models for Basic and Preclinical Research

**DOI:** 10.3390/cancers12092651

**Published:** 2020-09-17

**Authors:** Sirin Saranyutanon, Sachin Kumar Deshmukh, Santanu Dasgupta, Sachin Pai, Seema Singh, Ajay Pratap Singh

**Affiliations:** 1 Cancer Biology Program, Mitchell Cancer Institute, University of South Alabama, Mobile, AL 36604, USA; ss1830@jagmail.southalabama.edu (S.S.); skdeshmukh@health.southalabama.edu (S.K.D.); dasgupta@southalabama.edu (S.D.); seemasingh@health.southalabama.edu (S.S.); 2 Department of Pathology, College of Medicine, University of South Alabama, Mobile, AL 36617, USA; 3 Department of Medical Oncology, Mitchell Cancer Institute, University of South Alabama, Mobile, AL 36604, USA; spai@health.southalabama.edu; 4 Department of Biochemistry and Molecular Biology, University of South Alabama, Mobile, AL 36688, USA

**Keywords:** prostate cancer, research model, oncogenes, tumor suppressor genes

## Abstract

**Simple Summary:**

The molecular progression of prostate cancer is complex and elusive. Biological research relies heavily on in vitro and in vivo models that can be used to examine gene functions and responses to the external agents in laboratory and preclinical settings. Over the years, several models have been developed and found to be very helpful in understanding the biology of prostate cancer. Here we describe these models in the context of available information on the cellular and molecular progression of prostate cancer to suggest their potential utility in basic and preclinical prostate cancer research. The information discussed herein should serve as a hands-on resource for scholars engaged in prostate cancer research or to those who are making a transition to explore the complex biology of prostate cancer.

**Abstract:**

We have witnessed noteworthy progress in our understanding of prostate cancer over the past decades. This basic knowledge has been translated into efficient diagnostic and treatment approaches leading to the improvement in patient survival. However, the molecular pathogenesis of prostate cancer appears to be complex, and histological findings often do not provide an accurate assessment of disease aggressiveness and future course. Moreover, we also witness tremendous racial disparity in prostate cancer incidence and clinical outcomes necessitating a deeper understanding of molecular and mechanistic bases of prostate cancer. Biological research heavily relies on model systems that can be easily manipulated and tested under a controlled experimental environment. Over the years, several cancer cell lines have been developed representing diverse molecular subtypes of prostate cancer. In addition, several animal models have been developed to demonstrate the etiological molecular basis of the prostate cancer. In recent years, patient-derived xenograft and 3-D culture models have also been created and utilized in preclinical research. This review is an attempt to succinctly discuss existing information on the cellular and molecular progression of prostate cancer. We also discuss available model systems and their tested and potential utility in basic and preclinical prostate cancer research.

## 1. Introduction

Prostate cancer (PCa) is the most commonly diagnosed malignancy and the second leading cause of cancer-related death in men in the United States. It is estimated that PCa will afflict approximately 191,930 men and cause nearly 33,330 deaths this year in the United States alone [1]. Notably, PCa incidence and associated mortality are nearly two-thirds and over two times higher, respectively, in African-American (AA) men compared to their Caucasian-American (CA) counterparts [2,3]. PCa follows a defined pattern of cellular progression but exhibits diverse molecular pathobiology making it one of most highly heterogeneous cancers [4,5]. The prostate-specific antigen (PSA) test is the primary detection tool for PCa screening. However, due to the lack of accuracy and specificity, the usefulness of PSA for PCa diagnosis has been questioned [6,7,8]. Most PCa patients are generally subjected to localized radical prostatectomy, radiation therapy, proton beam therapy, and cryosurgery after the initial diagnosis [9,10,11]. However, for patients with metastatic disease or recurrent cancer with locoregional and distant metastases, androgen-deprivation therapy (ADT) or castration therapy is considered the primary line of treatment [12]. Unfortunately, despite the initial outstanding therapeutic response, most PCa patients treated with ADT eventually have the relapse of PCa in a highly aggressive and therapy-resistant form leading to poor clinical outcomes [13,14].

To meet the challenges associated with prostate cancer clinical management, research labs across the world have been working tirelessly to understand underlying molecular diversity and biology of PCa. These efforts have resulted in novel therapies that are currently in clinics, while researchers continue to gather more insights to address new hurdles and failures faced in clinical settings. These advances have been possible through the development of several in vitro and in vivo research models, while new models continue to be developed to address the genetic and biological complexities associated with the PCa. In this review, we discuss the cellular and molecular progression of PCa as well as the available in vitro and in vivo models for PCa research. We believe that the information presented herein will be helpful to the researchers, especially those who are new to the field, in understanding the molecular pathobiology of PCa and guide them in choosing the correct model(s) for their laboratory and preclinical research.

## 2. Cellular and Molecular Progression of Prostate Cancer

**Figure 1:**
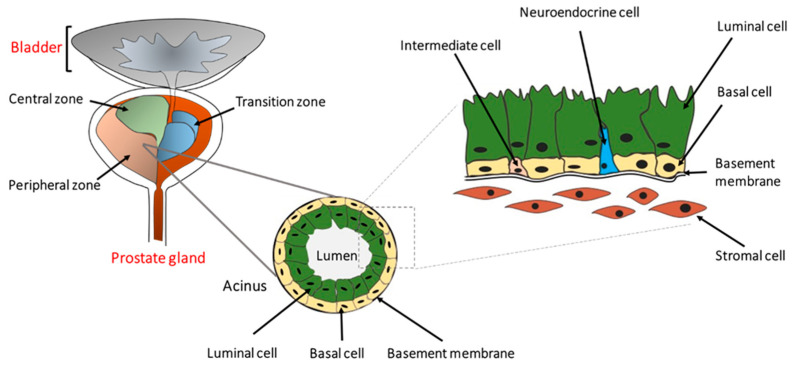
The location and architecture of the human prostate gland. The prostate gland is located below the bladder and consists of a central, a peripheral, and a transition zone. Histologically, it is comprised of secretary luminal, basal, and rare intermediate and neuroendocrine cells. The prostatic epithelium is separated from the stromal cells by the basement membrane as indicated. Preneoplastic or neoplastic cellular transformation can initiate from either basal or luminal cells.

The human prostate is a walnut-size glandular organ that develops from the embryonic urogenital sinus [15]. Its primary function is to produce seminal fluid containing zinc, citric acid, and various enzymes, including a protease named prostate-specific antigen (PSA). Histologically, the prostate can be divided into central, peripheral, and transition zones comprised of a secretory ductal-acinar structure located within a fibromuscular stroma [16,17]. The ductal-acinar structure is formed of tall columnar secretory luminal cells, a flattened basal epithelium attached to the basement membrane, and scattered neuroendocrine cells (Figure 1). Luminal epithelial cells express cytokeratins (CK) 8 and 18, NKX3.1, androgen receptor (AR), and PSA, whereas basal epithelial cells express CK5, CK14, glutathione S-transferase Pi 1 (GSTP1), p63, and low levels of AR [18,19].

**Figure 2:**
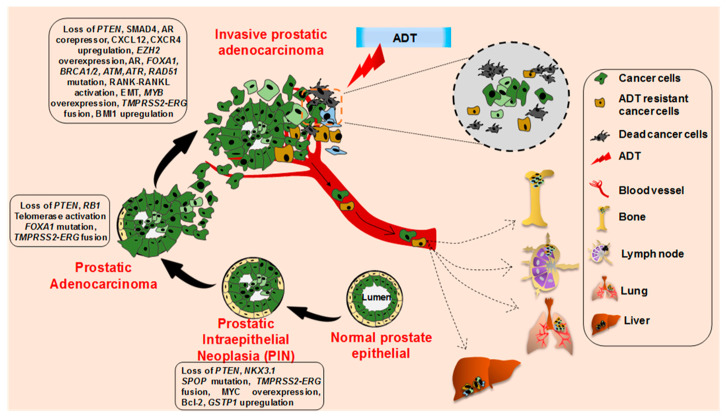
Histopathological and molecular progression of human prostate cancer. Metastatic prostate cancer develops via progression through prostate intraepithelial neoplasia (PIN) and invasive adenocarcinoma through the acquirement of various molecular alterations as depicted. The invasive adenocarcinoma cells and androgen-deprivation therapy resistant cancer cells metastasize to the bone, lymph node, lung, and liver.

The cellular origin of prostate cancer is not very clear, partly because of the lack of well-characterized prostate epithelial lineage [20,21,22]. PCa develops from normal prostate epithelium through a multistep histological transformation process, governed by various underlying molecular changes [23] (Figure 2). Low-grade and high-grade prostate intraepithelial neoplasia (PIN) lesions develop from normal prostate epithelium through the loss of phosphatase and the tensin homolog *(PTEN)*, NK3 Homeobox 1 (*NKX3.1)*, overexpression of *MYC* proto-oncogene, B-cell lymphoma 2 (*BCL-2),* and the glutathione S-transferase pi 1 gene (*GSTP1),* accompanied with Speckle Type BTB/POZ Protein (*SPOP)* mutation and Transmembrane Serine Protease 2- ETS-related gene (*TMPRSS2-ERG)* fusion [24,25,26,27,28,29,30,31,32,33,34,35,36]. Further loss of the retinoblastoma protein (*RB1)*, along with telomerase activation and frequent Forkhead Box A1 (*FOXA1)* mutation, leads to the development of prostate adenocarcinoma from the advanced PIN lesion [37,38,39,40,41,42,43]. Further molecular aberrations including the loss of SMAD Family Member 4 (*SMAD4)*, AR corepressors, mutations in AR, *FOXA1, BRCA1/2, ATM, ATR,* and *RAD51* accompanied with the gain of function of the AR coactivator, *CXCL12, CXCR4, RANK-RANKL,* EMT, *BAI1*, and *EZH2* lead to the development of metastatic prostate cancer [44,45,46,47,48,49,50,51,52,53,54,55,56,57,58,59].

As evident from the PCa progression model (Figure 2), inactivation of *PTEN* appears to be a critical event in PCa carcinogenesis and associated with aggressive disease manifestation. *PTEN* alterations occur in various ways in prostate cancer, such as genomic deletion and rearrangement, intragenic breakage, or translocation. The loss of *PTEN* is linked with an upregulation of PI3K/AKT/mTOR signaling that regulates cell survival, proliferation, and energy metabolism [60,61]. Another critical determinant of PCa tumorigenesis is *SMAD4*, a tumor suppressor gene (18q21.1), which mediates the transforming growth factor β (TGF-β) signaling pathway and suppresses epithelial cell growth. Transcriptome analysis revealed significantly lower levels of *SMAD4* in PCa tissues compared to adjacent non-cancerous tissues [46]. Of note, in a mouse model, prostate specific ablation of *Smad4* and *Pten* leads to the development of an invasive and metastatic potential of PCa (discussed below) [45].

In the PCa initiation and progression cascade, tumor suppressor *NKX3.1* (8p21) plays a pivotal role and found to be frequently lost due to the loss of heterozygosity (LOH) [62,63]. Of note, LOH at 8p21 appears to be an early event in PCa tumorigenesis [63,64,65]. Thus, it is likely that the genes that reside within these frequently deleted regions are associated with PCa initiation. Under the normal condition, *NKX3.1* drives growth-suppressing and differentiating effects on the prostatic epithelium [66]. *Nkx3.1* heterozygous mice develop abnormal prostate morphology with the dysplastic epithelium [67,68]. Importantly, *Nkx3.1*-null mice show changes in prostate epithelial morphology with severe dysplasia [67]. Kim et al. demonstrated that the loss of function of *Pten* and *Nkx3.1* in mice cooperated in PCa development. Importantly, *Pten;Nkx3.1* compound mutant mice showed a higher incidence of High-grade prostatic intraepithelial neoplasia (HGPIN) [69]. In addition to the critical tumor suppressor genes described above, the *MYC* proto-oncogene is also amplified in PCa [70,71,72]. *MYC* encodes a transcription factor that regulates the expression of several genes involved in cell proliferation, metabolism, mitochondrial function, and stem cell renewal [73,74,75]. Several studies suggest that *MYC* is activated through overexpression, amplification, rearrangement, Wnt/β-catenin pathway activation, germline *MYC* promotor variation, and loss of *FOXP3* in PCa [76,77,78,79], and is a critical oncogenic event driving PCa initiation and progression [71,80].

Other than *MYC, TMPRSS2:ERG* gene fusion, resulting from the chromosomal rearrangement, is also reported in approximately 45% of PCa. This alteration leads to the expression of the truncated *ERG* protein under the control androgen-responsive gene promoter of *TMPRSS2* [81,82,83,84,85]. *ERG* belongs to the *ETS* family of transcription factors (*ERG, ETV1*, and *ETV4*), and its activation is associated with PCa progression in both early- and late-stages [82,83,86]. *MYB*, another gene encoding a transcription factor, is also reported to be amplified in PCa and exhibits an increased amplification frequency in castration resistant PCa (CRPC) [87]. Research from our laboratory has shown that *MYB* plays a vital role in PCa growth, malignant behavior, and androgen-depletion resistance [56].

## 3. Prostate Cancer Research Models

As discussed above, we have made appreciable progress in our understanding of PCa pathobiology over the past several years. These insights resulted from the efforts at multiple levels: (i) recording of clinicopathological data and histopathological examination of tumor sections at the microscopic levels, (ii) molecular profiling of clinical specimens to identify molecular aberrations associated with defined histopathological characteristics, and (iii) conducting laboratory assays to define the functional significance of identified molecular aberrations. The development of PCa research models by scientists played a significant role in these laboratory and preclinical efforts. Prostate cell lines (cancer and non-cancer) established from patients have been instrumental as research models to gain functional and mechanistic insight. A comprehensive list of cell lines used in PCa research is given in Table 1. Moreover, quite a few mouse models have also been developed that not only provide direct evidence for the oncogenic function of a gene or gene-set but also serve as models for furthering basic and translational cancer research. Recently, 3-D in vitro cultures and patient-derived tumor xenografts (PDXs) have been developed as well, which are mostly used for translational research. Below we describe some of these models and discuss their characteristics and potential significance.

### 3.1. Cell Line Models

#### 3.1.1. Non-Cancerous Prostate Epithelial Cell Lines

##### RWPE-1

This cell line model was established from the peripheral zone of a histologically normal adult human prostate from a 54-year-old man. The cells were immortalized by transduction with human papillomavirus 18 (HPV-18) to establish a stable line [88]. RWPE-1 cells exhibit the expression of AR and androgen-inducible expression of kallikrein-3 (KLK3) or PSA. These cells also express CK8 and CK18, which are the characteristic markers of the luminal prostatic epithelium [89]. Further, RWPE-1 cells exhibit heterogeneous nuclear staining for p53 and Rb proteins as well [89]. The growth of these cells is induced upon treatment with the epidermal growth factor (EGF) and fibroblast growth factor (FGF) in a dose-dependent manner, whereas TGF-β treatment inhibits their growth [89,106,107].

##### BPH-1

BPH-1 is an immortalized benign prostatic hyperplasia cell line model established from primary prostatic tissue obtained by transurethral resection from a 68-year-old patient [90]. Immortalization of these cells was achieved by transduction with simian virus 40 (SV40) large T antigen [90]. BPH-1 cells express wild type (WT) PTEN, WT p53 as well as CK8, CK18, and CK19 suggestive of their luminal epithelial origin [108], but are negative for AR, PSA, and prostatic acid phosphatase (PAP) [90]. Cytogenetic analysis of these cells revealed an aneuploidy karyotype with a modal chromosome number of 76 (range 71-79). EGF, TGF-β, FGF-1, and FGF-7 treatment induces the proliferation of these cells, while FGF-2, TGF-β1, and TGF-β2 are shown to have an opposite effect [90]. Due to the lack of AR expression, these cells do not respond to androgen treatment [90]. They are non-tumorigenic in nude mice [108].

##### pRNS-1-1

pRNS-1-1 is a human prostatic epithelial cell line model derived from a 53-year-old male who had undergone radical prostatectomy. These cells were transfected with a plasmid, pRNS-1-1, containing the SV40 genome expressing T-antigen to establish a stable line. pRNS-1-1 cells express WTPTEN, and CK5 and CK8 suggestive of their epithelial origin [91]. The pRNS-1-1 cells do not express either AR or PSA [109,110]. The growth of these cells is promoted by EGF, IGF, and bovine pituitary extract treatment, while TGF-β has an inhibitory effect. pRNS-1-1 cells do not form tumors when injected subcutaneously in nude mice [109].

##### RC-77N/E

The RC-77N/E prostate epithelial cell line model was derived from the non-malignant prostate tissue isolated from a 63-year-old African American (AA) man diagnosed with PCa [92]. RC-77N/E cells are immortalized by the expression of HPV-16E6/E7 and exhibit an epithelial morphology. These cells are androgen-sensitive and express CK8, AR, PSA, and p16. RC-77N/E does not form tumors in severe combined immunodeficiency (SCID) mice [92]. This line could be useful for racial disparity associated PCa studies.

##### HprEpC

Human prostate epithelial cells (HprEpCs) were isolated from the normal human prostate. HPrEpC model cells express both prostatic basal epithelial marker CK14 and luminal prostatic epithelium markers CK18 and CK19 suggesting that they are intermediate cells [93]. Besides their application as normal control cells for PCa research, HprEpC cells are useful tools in studying the hormonal regulation and secretory function of the prostate.

#### 3.1.2. Prostate Cancer Cell Lines

Prostate cancer cell lines established from human patients are broadly categorized into two types (castration-sensitive and castration-resistant) depending upon their survivability under androgen-deprived conditions.

##### Castration-Sensitive

###### LNCaP

LNCaP is a widely used human PCa cell line model. This cell line was developed in 1980 from a lesion in the left supraclavicular lymph node metastasis of human prostatic adenocarcinoma from a 50-year-old Caucasian male [94]. LNCaP cells are weakly adherent and slow-growing and have a doubling time of about 60-72 h. LNCaP cells express AR and PSA and exhibit a biphasic regulation of growth following androgen treatment [111]. These cells have a point mutation in AR (T877A) and express WT p53 [112,113]. These cells also harbor one mutated and other deleted alleles of *PTEN* [114]. Additionally, these cells are CK8, CK18, CK20, and vimentin-positive [115]. LNCaP cells require androgens to sustain their growth, but several derivative androgen-depletion resistant cell lines have been developed following slow and long-term androgen-deprivation or through their selection from mouse-xenograft tumors [116,117].

###### LAPC-4

LAPC-4 (Los Angeles prostate cancer 4) model cell line was established from a lymph node metastasis of a hormone-refractory PCa patient through direct transfer of surgically removed tissues (2–3 mm sections) into male SCID mice. The tissue explants were subcutaneously xenografted into the mice, and later tumor cells were harvested from mouse xenografts and plated on the culture dish to generate the cell line [95]. These cells are very slow growing, with a doubling rate of around 72 h [113]. LAPC-4 cells express wild type AR and PSA [118]. The expression of both CK5 (a basal epithelial marker) and CK8 (luminal epithelium marker) is also detected in these cells suggestive of their dedifferentiation [95]. Although these cells are castration-sensitive, forced overexpression of human epidermal growth factor receptor 2 (HER-2/neu) is shown to cause ligand independence by activation of the AR pathway [119]. Further, HER2 overexpression synergizes with low levels of androgen to potentiate AR activation [119]. LAPC4 are tumorigenic and can grow subcutaneously, orthotopically, or intratibially in nude mice [120,121,122].

###### LAPC-9

The LAPC-9 (Los Angeles prostate cancer 9) cell line was derived from the bone metastasis of the prostate cancer patient that had undergone androgen-ablation therapy [96]. These cells express AR and PSA and undergo growth arrest upon androgen ablation [123]. It is shown that LAPC-9 cells can remain in a dormant state for at least six months following castration and can emerge as castration-resistant following a long period of androgen deprivation [96]. LAPC-9 cells develop tumors in nude mice upon subcutaneous injection [96,124]. They can respond rapidly to androgen replenishment and re-enter the cell cycle and resume growth [96].

###### RWPE-2

The RWPE-2 cell line is derived from the HPV-18 immortalized RWPE-1 cells by transformation with Ki-ras using the Kirsten murine sarcoma virus (Ki-MuSV). The overexpression of Ki-ras bestowed tumorigenicity to these cells since Ki-ras activation is implicated in prostate carcinogenesis [89]. These cells express CK8, CK18, WT p53, WT Rb, AR, and PSA and are hormone responsive. EGF and FGF promote RWPE-2 cell growth, and in contrast, TGF-β has growth inhibitory effects on these cells. RWPE-2 cells that form colonies in agar have an invasive potential [89] and form tumors when injected subcutaneously into the nude mice [125].

###### VCaP

The VCaP (vertebral cancer of the prostate) cell line was established in 1997 from a metastatic prostate tumor that developed in the vertebrae of a 59-year-old Caucasian patient with the hormone-refractory disease who had failed androgen deprivation therapy [97]. VCaP was passaged as xenografts in nude mice and then cultured in vitro. The VCaP cells exhibit multiple features of clinical PCa, including expression of PSA, PAP, and AR. One study has also shown the elevated expression of the AR-V7 variant in VCaP xenograft after castration by next-generation RNA-Seq [126]. Additionally, these cells express CK-8, CK-18, Rb, and p53 (with A248W mutation). As per the American Type Culture Collection, the doubling time of this cell line was about 51 h (VCaP ATCC CRL-2876TM). These cells form tumors when injected subcutaneously in SCID mice [97,127]. The presence of the *TMPRSS2:ERG* fusion gene has been shown to stimulate the growth of the VCaP orthotopic mouse model [128].

###### MDA-PCa 2a/2b

MDA-PCa 2a and MDA-PC 2b cell lines were established from two distinct areas of prostate tumor derived from a 63-year-old African American (AA) subject having a late-stage bone metastasis [98]. The patient was under relapse following castration therapy at the time of cell isolation. MDA-PCa 2a/2b cells express WT AR, WT p53, KLK3/PSA, WT PTEN, and p21 [129,130]. Coming from two different areas of the tumor, they have different doubling times. MDA-PCa 2a cells double in number in about 82–93 h, whereas MDA-PCa2b has a doubling time of 42–73 h. [98]. These cells can form tumors in mice when injected subcutaneously [98]. Although, the MDA-PCa 2a/2b cells are derived from an androgen-independent tumor but are sensitive and responsive to androgens [98]. Among these lines, MDA-PCa 2b is androgen dependent [131]. Later, a new androgen refractory subline MDA-PCa 2b-hr was developed following 35 weeks of androgen depletion to represent clinical PCa recurrence during androgen ablation treatment [131]. These lines could also be useful for racial disparity-associated PCa studies.

###### LuCaP 23.1

LuCaP 23.1, Lucan 23.8, and LuCaP 23.12 cell line series were developed in 1996 from two different lymph node metastases (LNM) of a 63-year-old Caucasian PCa patient (adenocarcinoma with Gleason score 8). Cancer tissues from this subject were xenografted subcutaneously in nude mice and passaged serially to establish these xenograft lines. All three lines are AR-positive and responsive to androgen and express WT PTEN at mRNA levels [99]. Notably, androgen depletion in mice harboring these three lines prolonged tumor growth with a concomitant decrease in the PSA expression level. However, some of the tumors eventually relapsed following castration and were considered hormone-refractory. Thus, studying these models could be invaluable to unravel the sequential molecular events driving relapse and acquirement of androgen independence. Moreover, tumor progression in these models can be monitored by measuring the PSA level. The LuCaP 35 model was developed from the LNM of a 66-year-old PCa patient (Stage T4c) through subcutaneous implantation in nude mice, as described above. This line expresses PSA and AR (harbors AR amplification and C1863T mutation) and is androgen-sensitive [132]. The LuCaP 35 cells can be cultured in vitro, unlike the LuCaP 23 cells, and produce LN and pulmonary metastases when implanted orthotopically. The LuCaP 35V cells were established from recurrent LuCaP 35 cells and are androgen-independent. Collectively, these are unique in vivo and in vitro models to study the mechanism of castration resistance. [133]. Later, several cell lines such as LuCaP 23.12, LuCaP 23.8, LuCaP 35, LuCaP 41, LuCaP 49, LuCaP 58, and LuCaP 73 were developed. LuCaP 23.1, LuCaP 23.12, LuCaP 23.8, LuCaP 35, LuCaP 41, LuCaP 49, LuCaP 58, and LuCaP 73 cells express AR and PSA.

###### RC-77T/E

The RC-77T/E cell line was developed from the radical prostatectomy specimen of a 63-year-old AA patient with a clinical-stage T3c adenocarcinoma [92]. From the same patient, anon-malignant cell line RC-77N/E was also developed (discussed above). The RC-77T/E cells express AR, PSA, NKX 3.1, CK8, and p16 [92]. RC-77T/E cells also express β-catenin, α-actinin-1, and filamin-A [134]. These cells are androgen-responsive and form tumors when injected subcutaneously in nude mice [92]. This cell line model could be useful for racial disparity-associated PCa studies.

###### 12T-7f

12T-7f (12: 12 kb, T: Tag transgene, f: fast) is a mouse cell line developed from the probasin-large T antigen transgenic mouse (a.k.a LADY) model along with six other transgenic cell lines. These cells were split into three groups based on the stage of neoplasia and their rapid growth pattern. Inoculation of these cells in mice resulted in the development of prostate tumors. The most aggressive line from these pools was designated as 12T-7f, which could progress to late-stage adenocarcinoma [135]. Notably, tumors developed through 12T-7f xenografting regressed upon castration but progressed after androgen administration.

##### Castration-Resistant Cell Lines

As discussed in the earlier section, castration-resistance could develop due to AR-dependent and AR-independent mechanisms. Therefore, two types of castration-resistant cell lines (AR-positive and AR-negative) have been developed and are discussed below:

###### Androgen-Receptor Expressing

####### C4-2/C4-2B

These cell lines were derived from LNCaP mouse xenografts. C4-2 was isolated from the vertebral metastasis of the LNCaP xenograft, whereas C4-2B was derived from the bone metastasis of the C4-2 tumor-bearing mice [102,103]. Both cell lines express AR and PSA and low levels of p53 and develop tumors when subcutaneously injected in the nude mice [103].

####### 22. Rv1

The 22Rv1cell line was introduced in 1999. This cell line was derived from the mouse CWR22R xenograft developed from the prostate tumor of a patient with bone metastasis [104]. The 22Rv1 cells harbor the H874Y mutation in the AR like CWR22R xenograft and express PSA and kallikrein-like serine protease [104,136]. EGF is shown to promote the growth of 22Rv1 in vitro [104]. Recently, it has been shown that 22Rv1 prostate carcinoma cells produce high-titer of the human retrovirus XMRV (xenotropic murine leukemia virus-related virus) [137].

###### Androgen-Receptor Non-Expressing

####### PC-3

The PC-3 cell line was developed from lumbar vertebral metastasis of a grade IV prostatic adenocarcinoma from a 62-year-old Caucasian man [100]. In the karyotypic analysis, these cells were found to be near triploid having 62 chromosomes. PC3 cells express CK7, CK8, CK18, and CK19 but not AR and PSA and exhibit characteristics of a poorly differentiated adenocarcinoma with a doubling time of about 33 h [138,139]. These cells respond positively to EGF while being insensitive to FGF and are tumorigenic when orthotopically injected in mice [100,140,141,142,143].

####### DU-145

The DU145 cell line was established from the brain metastasis of a 69-year-old prostate cancer patient [101]. These cells express CK5, CK7, CK8, CK18, and CK19 [93,144,145]. Being AR negative, DU145 cells are hormone-insensitive and do not express PSA [146]. This cell line has a doubling time of about 34 h and exhibits a growth response to EGF [147] and also a high level of EGFR expression [148]. DU-145 cells metastasize to spleen and liver when injected subcutaneously in a nude mouse. [149,150].

####### ARCaP

ARCaP (androgen-refractory cancer of the prostate) was established from the ascites of a patient with advanced metastatic disease. Interestingly, it is shown that androgen and estrogen treatment as a dose-dependent suppressive impact on the growth of ARCaP cells [105]. ARCaP cells express low levels of AR and PSA and exhibit positive immunostaining for EGFR, HER2/neu, HER3, bombesin, serotonin, neuron-specific enolase, and the mesenchymal–epithelial transition factor (C-MET). These cells are tumorigenic and highly metastatic that preferably colonize to the lung, pancreas, liver, kidney, and bone [151,152,153]. These cells form ascites fluid in athymic mice [105].

### 3.2. Genetically Engineered Mouse Models of Prostate Cancer

The mouse models are beneficial resources to improve our understanding of the disease pathobiology and to establish the role of candidate oncogenes in the pathogenic processes. As discussed below, several genetically engineered mouse models of PCa have been developed that have provided insights into tumor initiation, progression, and metastasis and are being used in preclinical research.

#### 3.2.1. TRAMP

The transgenic adenocarcinoma of the mouse prostate (TRAMP) mice model was generated and characterized in 1996. The chloramphenicol acetyltransferase (CAT) gene was introduced into the germ line of mice under the control of the rat probasin (PB) promoter. In TRAMP mice, expression of both the large and small SV40 T antigens (TAG) is regulated by the prostate-specific rat PB promoter [154]. The PB-SV40 T antigen (PB-Tag) transgene is spatially restricted to the dorsolateral and ventral lobes of the prostate. The gene expression is male specific and restricted to the epithelial cells of the lateral, dorsal, and ventral prostatic lobes of the murine prostate [155]. TRAMP is a very useful model for studying the pathology of PCa as the progression occurs through PIN lesions to malignant disease, like human disease, in a predictable time. Epithelial hyperplasia develops by 10 weeks of age, PIN by 18 weeks of age, and lymphatic metastases after 28 weeks of age [154,156,157].

The TRAMP model has been used for PCa prevention and treatment studies [158,159]. It is also the first genetically engineered mouse model (GEMM) that displays castration-resistant disease progression [160]. One of the limitations of the TRAMP model, however, is that these mice often develop neuroendocrine PCa [161]. A simultaneous loss of *Rb* and *p53* could be the reason for the development of neuroendocrine cancer [161,162]. Considering the higher chances of neuroendocrine disease, the TRAMP mouse model is clinically more relevant to study PCa of neuroendocrine origin.

#### 3.2.2. LADY

The LADY PCa mouse model was developed in 1998 and is similar to the TRAMP model [163]. There are, however, a few key differences between the TRAMP and LADY. In the LADY, a larger fragment (12 kb) of the PB (a.k.a. LPB) promoter upstream of the SV40 T-antigen is used that contains additional androgen and growth factor-responsive sequences and thus allows consistently high transgene expression. Additionally, the LPB promoter is linked with a deletion mutant of the SV40 T-antigen (deleted small T-antigen) to allow the expression of large T-antigen, unlike small t-antigen in the TRAMP model. The purpose of deleting small t-antigen was to analyze the importance of neuroendocrine differences in metastatic lesions developed by LADY [164]. LADY model mice develop metastases to the liver, lymph nodes, and bones [164]. The metastases, however, primarily contain neuroendocrine cells, which is unlike the human metastasis [135,165]. Thus, the LADY mice are different from the most common type of human PCa from the perspective of rapid tumor growth and neuroendocrine tumor development. Nevertheless, the LADY model possesses the molecular changes similar to the human prostate, such as the multifocal nature of tumorigenesis, histopathologically changes from low- to high-grade dysplasia similar to PIN in humans, and the androgen-dependent growth of the primary tumors. Hence, the LADY model could be beneficial for investigating the stepwise mechanisms of PCa progression as well as therapeutic intervention [163].

#### 3.2.3. Pten Deficient Mice

Loss of the *PTEN* tumor suppressor is a critical event in PCa initiation, as discussed above. However, homozygous knockout of *Pten* in mice embryonic stem cells through the deletion of the phosphatase domain led to embryonic lethality [166,167]. To overcome this limitation, Wang et al. generated *Pten* null mice by conditional deletion of *Pten* in the murine prostatic epithelium. They generated *Pten* ^loxp/loxp^: PB-Cre4 mice in order to attain the prostate-specific *Pten* biallelic deletion. They showed that *Pten* null PCa progressed with a short latency of PIN formation by 6 weeks of age compared to heterozygous *Pten* deletion mice, which developed PIN by 10 months. Moreover, homozygous *Pten* deletion mice developed invasive adenocarcinoma by 9 weeks of age and metastasis to the lymph node and lung by 12 weeks of age. The effect of hormone ablation therapy on *Pten* null mice was evaluated by performing the castration of mice at week 16. The response of *Pten* null tumors at day 3 and day 6 post-castration was analyzed. In response to androgen abolition, the AR-positive prostatic epithelium showed an increase in the apoptosis leading to the decrease of prostate volume. Hence, these homozygous *Pten* mutant mice recapitulate the PCa by mimicking the histopathological features of human disease [40]. In contrast, heterozygous mutant *(Pten^+/−^)* mice developed neoplasia in multiple tissues, including mammary glands, lymphoid cells, small intestines, thyroid, endometrial, and adrenal glands [166,168,169], further limiting the applicability of the heterozygous mutant over *Pten* null mice.

The *Pten* knockout model has been used to demonstrate the role of the tumor microenvironment, particularly interleukin-17 (IL-17), in the growth and progression of PCa [170,171]. To test how tumor suppressor *Rb* interacts with *Pten*, Bai et al. developed mice with double mutations in both the cyclin-dependent kinase (CDK) inhibitor *p18Ink4c* and *Pten* [172]. The double mutant mice develop a broader spectrum of prostate tumors in the anterior and dorsolateral lobes at an accelerated rate [172]. Loss of function of *Nkx3.1* is crucial for PCa progression and has been associated with the development of prostatic epithelial hyperplasia, dysplasia, and PIN [30,67,173]. *Nkx3.1* and *Pten* are shown to cooperate in prostate carcinogenesis in mice. *Nkx3.1;Pten* double mutant mice demonstrated an increased incidence of HGPIN, which resembles the early stages of human PCa [69].

#### 3.2.4. Pten^pc−/−^Smad4^pc−/−^

To examine a cooperative action of *Pten* and *Smad4* loss in PCa pathogenesis, De Pinho lab developed mice having prostate-specific genetic ablation of *Smad4* in Pten-null mice. These mice were highly aggressive and exhibited profound lymph node and pulmonary metastasis [45]. The importance of *Smad4* in PCa was further revealed by the development of metastatic and lethal PCa with 100% penetrance in *Smad4* and *Pten* double knockout mouse prostate [45]. *Pten^pc−/−^Smad4^pc−/−^* has been used to analyze the efficacy of hypoxia-prodrug TH-302 and checkpoint blockade combination therapy. The combination of the hypoxia-prodrug and checkpoint blockade significantly extended the survival of *Pten^pc-/-^Smad4^pc-/-^* mice [174]. Furthermore, Wang and colleagues utilized the *Pten^pc−/−^Smad4^pc−/−^* mice model and identified that polymorphonuclear myeloid-derived suppressor cells (MDSCs) are one of the significant infiltrating immune cells in PCa and their depletion blocks PCa progression [175].

#### 3.2.5. Hi/Lo-Myc

Two plasmids having a rat probasin (PB) promoter alone (PB-Mycfor lo-Myc) and PB coupled with a sequence of the ARR2 (ARR2PB for hi-Myc) were used to achieve prostate-specific overexpression of c-Myc. The ARR2PB promotor contained two additional androgen response elements that forced the development of invasive adenocarcinoma from prostatic intraepithelial neoplasia (mPIN) in about 26 weeks [27,176,177]. Hi-Myc mice also displayed a decreased expression of Nkx3.1 at both mRNA and protein levels [27]. The PB-Myc mice showed similar pathological changes, but a slower progression of 30 weeks (time to invasive PCa development from PIN lesions) [27]. The main differences between these two models are their androgen responsiveness. The Hi-Myc is androgen-responsive, while the Lo-myc model displays no such sensitivity [27]. The mice model generated by non-viral oncogene ARR2PB-Myc and PB-Myc develop invasive adenocarcinoma and offer advantages over those expressing SV40. However, they do not develop metastasis, which is a major drawback of this model. Hubbard et al. in 2016 showed that the combination of *Myc* overexpression and *Pten* loss in mice resulted in the development of lethal prostatic adenocarcinoma with distant metastases [29]. Moreover, homeobox protein Hox-B13 (HOXB13) was suggested to participate in the *MYC* activation and *Pten* loss genomic instability and aggressive prostate cancer [29,178].

#### 3.2.6. MPAKT

The mouse prostate Akt (MPAKT) model is useful in studying the role of protein kinase B (Akt) in the transformation of prostate epithelial cells and in developing the biomarkers relevant to human PCa. This mouse model was developed by the introduction of Akt1 along with a myristoylation sequence (myr) and a hemagglutinin (HA) epitope in the form of the linearized rPb-myr-HA-Akt1. This insert was injected into the pronuclei of fertilized oocytes, and the friend leukemia virus B (FVB) mice founders were verified [179]. These mice exhibited the formation of PIN by 8 weeks. Immunohistochemistry analysis of the PIN lesions of MPAKT demonstrated numerous important findings such as Akt results in the activation of p70S6K and is associated with the development of PIN in MPAKT mice and Akt-induced PIN might be linked to neovascularization. Histological evaluation revealed that MPAKT mice had distinct phenotypic characteristics, including disorganized epithelial layers, loss of cell polarity, intraepithelial lumen formation, and nuclear atypia and apoptotic bodies. However, the MPAKT did not develop invasive carcinoma even after 78 weeks [180].

### 3.3. Patient Tumor-Derived Models

Patient-derived models are useful tools for translational research as they mimic human tumors. They are instrumental in studying the response of various therapies undergoing preclinical evaluation since they carry intrinsic tumor factors and microenvironmental presence involved in disease progression and therapy resistance.

#### 3.3.1. Three-Dimensional (3-D) Organoid Cultures

The transition from monolayer PCa cultures to the three-dimensional (3-D) cultures is a remarkable breakthrough in cancer research. Although culturing cancer cell lines is cost-effective and easy to handle, established cell lines do not carry the heterogeneity and genetic makeup of tumors from which they were initially derived [181,182]. These limitations are mostly overridden by the establishment of 3-D organoid culture models from the patient-derived tumors [183]. Dong et al. established the first PCa 3-D organoid culture from the biopsy of a patient in 2014 [184]. This organoid culture maintained the molecular signature of PCa, including *TMPRSS2-ERG* fusion, *SPOP* mutation, Chromodomain Helicase DNA Binding Protein 1 (*CHD1)* loss, and serine protease inhibitor Kazal-type 1 (*SPINK1)* overexpression. Further, whole-exome sequencing revealed mutations in several other genes, as well as the loss of the p53 and RB tumor suppressor pathway function [184]. Puca and colleges developed patient-derived organoids from needle biopsies of metastatic lesions from patients with neuroendocrine CRPC. These organoids showed genomic, epigenomic, and transcriptomic association with corresponding patient tumors [185]. 3-D models are thus beneficial for drug discovery and preclinical evaluation of therapeutic drugs for efficacy under in vitro setting that mimics the complex in vivo environment.

#### 3.3.2. Patient-Derived Xenografts (PDX)

Patient-derived xenografts (PDXs) are essential tools in cancer research as the results obtained from these resources more accurately predict clinical responses in patients (Table 2). The reason is that these models retain the genetic diversity of patient tumors and maintain a closely resembling tumor microenvironment [186]. PDX grown in immunocompromised mice carry essential histological and molecular features of the patient tumors, including gene expression programs, mutations, epigenetic regulators, and structural genomic events that ultimately drive their 3D growth [187,188]. Recent technical advancements, including the co-injection of PCa tissues with extracellular matrix (ECM) and transplantation into renal capsules, have increased the success rate of PDX establishment in mice [189,190,191]. The first androgen-dependent PCa xenograft model, designated as PC-82, was developed in 1977 by Schröder and colleagues at Erasmus University Rotterdam [192]. For this, the patient prostatic tumor tissue was grafted into the shoulder of nude mice. Later, two more androgen-independent in vivo models, designated as PC-133 and PC-135, were developed [192]. In 1996, seven other PDX models were established [193]. During 1991-2005, numerous other PDX models were developed that carried the *TMPRSS-ERG* rearrangement, *RB1* loss, AR amplification, *PTEN* deletion, *SPOP* mutation, *Tp53* deletion and mutation, and *BRCA2* loss [132,194,195]. The success rate of the localized PDX model has been increased in recent years due to the implantation of the chimeric graft with neonatal mouse mesenchyme. This method improved the survival rate and doubled the proliferation index of xenografted cancer cells [196]. The PDX models, however, have two significant limitations, i.e., the absence of functional human immunity and the lack of orthotopic modeling in the mice [197]. Further, the model takes a long time (about 8 months) for validation of detectable tumor growth in mice that limits its utility for the high-throughput drug screening [198].

### 3.4. Other Models

#### 3.4.1. Rat Models

Rat is one of the models for PCa research that was first established in the year 1937 by Moore and Melchionna after injecting the white rat prostate with benzpyrene. Following treatment, the columnar prostate epithelium underwent squamous metaplasia and also led to the induction of cancer in both the healthy and atrophic prostates [199]. These tumors spontaneously developed from a dorsal prostatic adenocarcinoma in an inbred Copenhagen rat and then were transplanted into a syngenic Copenhagen × Fischer F1 hybrid rat. These rat prostate tumors are well differentiated and slow growing [200]. The albino Lobund–Wistar (LW) rat model was first described by Pollard [201]. The LW rat developed spontaneous tumors at a mean age of 26 months. Moreover, a combination of N-methyl-N-nitrosourea (MNU) and testosterone treatments induced the development of prostate adenocarcinoma in the LW rat at a mean time of 10.5 months. The cancer of the LW rat resembles the human PCa in several aspects, including spontaneous development and progression to androgen independence and metastasis [201]. However, a major limitation of the rat models is that they have a long latency period for tumor development (2–3 years), have low tumor incidence, and lack spontaneous metastases.

#### 3.4.2. Zebrafish Model

The zebrafish model for cancer research has been utilized by many to acquire information that is traditionally obtained by mice and cell culture systems, although there are limited studies on zebrafish in an in vivo model for PCa research. The zebrafish model is suitable for visual observation of labeled tumor cells through the imaging technique since they are transparent. Nevertheless, the limitation of orthotopic transplantation could be the hurdle owing to the anatomical difference between zebrafish and the human body such as the breast, prostate, or lung [202]. The cancer cells can be injected into a different site in the zebrafish embryos, such as the blastodisc region, the yolk sac, the hindbrain ventricle, and into the circulation via the duct of Cuvier [203,204]. Melong et al. inoculated androgen-sensitive LNCaP cells into zebrafish and observed the effect of testosterone on the growth. Administration of exogenous testosterone increased the proliferation of PCa cells [205]. Further, the growth-promoting effect of testosterone was reversed by the anti-androgen receptor drug, enzalutamide. The invasive potential of PC3 cells overexpressing the calcitonin receptor (CTR) has also been evaluated in the zebrafish model [206]. The zebrafish model has several advantages, including the fact that zebrafish are small and can generate a large number of offspring in a short time, and they are easy to maintain and observe owing to their transparency. Moreover, humans and zebrafish have 71% protein similarity, and, most importantly, zebrafish absorb molecules from water providing an additional route for drug administration.

## 4. Conclusions and Future Outlook

**Figure 3:**
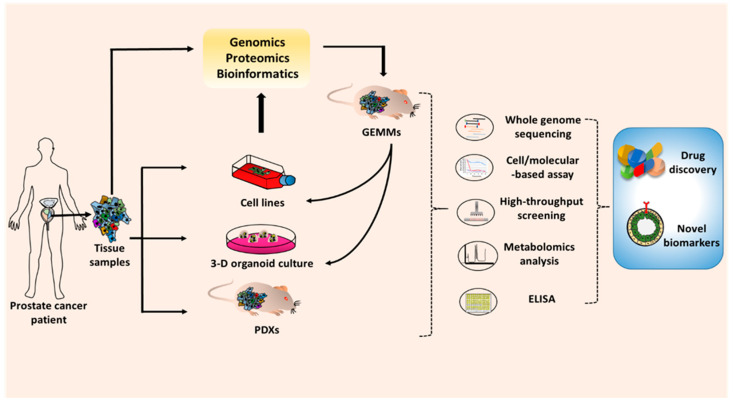
Application of the prostate cancer model in basic and preclinical cancer research. To develop the novel drugs or biomarkers, the prostate cancer models are required for in vitro and in vivo studies. The prostate cell lines, 3D-organiods, and patient-derived tumor xenografts (PDXs) can be generated from prostate tumor tissue from human patients. Patient tumor tissues can be also used to create genetically engineered mouse models (GEMMs). The results from research and preclinical studies are validated through several techniques such as whole genome sequencing, cell and molecular-based assays, high-throughput screening, metabolomics analysis, and ELISA. The promising drugs or biomarkers that emerge from those works will subsequently progress to preclinical and clinical studies.

In the past years, understanding of PCa pathobiology paired with mechanistic studies has remarkably advanced the field of PCa research. This insight has only been possible because of the availability of several types of research models. These models have been extremely helpful in improving our knowledge of PCa etiology, development, and metastatic progression. The cell line models have offered an easy and inexpensive platform to study the functions of aberrantly-expressed genes and various types of genetic alterations including gene mutations, splice variants, gene rearrangements, etc. Furthermore, cell lines serve as a primary model for screening of newer drugs or drug combination and provide us data on the molecular mechanisms of therapy resistance that is crucial for drug development. Since cell lines do not completely capture the tumor heterogeneity and are not grown in a complex microenvironment that tumor cells encounter in vivo, other in vivo models play an important role in further evaluation of gene functions and drug efficacies. The 3D-tissue culture model mimics the in vivo system under in vitro settings and has proven very useful in drug screening. Further, as the field of precision medicine is developing, these models could be of great significance in patient-tailored treatment planning based on preliminary assessment. Patient-derived xenografts (PDXs) grown in mice are useful as they more closely mimic a human tumor in vivo microenvironment. Genetically engineered mouse models (GEMs) are useful as they capture the complete progression of PCa from initiation to metastatic spread under a non-immunocompromised environment. Further, these models also develop a variety of PCa tumor types although they do not have the complete molecular diversity of human tumors (Figure 3). Regardless of limitations, each model has its own importance and these models often complement each other and are often utilized in progressive sets of experiments. There is, however, a need to develop models representing PCa of different racial and ethnic groups considering racial health disparities in incidence and clinical outcomes. Our refined knowledge of tumor genetics and awareness of health disparities and technologically advances will help us make further progress and we would continue to add to our list of PCa tumor models.

## Figures and Tables

**Table 1:** Prostate cancer cell line models and their characteristics.

Cell Line	Origin	Doubling Time	AR	PSA	Markers	Cyto-Keratin	Source	Refs.
Non-cancerous prostate epithelial cell lines								
RWPE-1	NPEC in peripheral zone	120 h	+	+	p53, Rb	8, 18	ATCC	[88,89]
BPH-1	Primary prostatic tissue	35 h	−	−	p53, BAX, PTEN, p21	8, 18, 19	ACCEGEN, Creative Bioarray, DSMZ	[90]
pRNS-1-1	radical prostatectomy	72 h	−	−	PTEN	5, 8	NCI and Stanford University	[91]
RC77N/E	Non-malignant tissue of a PCa patient	No report	+	−	NKX3.1, p16	8	Tuskegee University	[92]
HprEpC	Normal human prostate	No report	+	+	Cytokeratin 18	14, 18, 19	Cell applications, iXcells Biotechnologies, EZ biosystem	[93]
Hormone sensitive								
LNCaP	lymph node metastatic	28–60 h	+	+	WT p53, PTEN loss, vimentin, PAP, CBP, negative desmin	8, 18, 20	ATCC, Creative Bioarray, ACCEGEN, SIGMA	[94]
LAPC-4	lymph node metastatic from an androgen insensitive patient	72 h	+	+	p53 mutation	5, 8, 18	ATCC *	[95]
LAPC-9	bone metastasis from a patient with ADT	No report	+	+	Ki67, PTEN loss	5	ATCC *	[96]
VCaP	metastatic tumor	51 h	+	+	p53 mutation, Rb, PAP, PTEN	8, 18	ATCC, SIGMA, ACCEGEN	[97]
MDA-PCa 2a/2b	bone metastasis from an African-American male	82–93 h/42–73 h	+	+	WT p53, p21, Rb, Bcl-2	5, 8, 18	ATCC	[98]
LuCaP 23.1	lymph node and liver metastatic	11–21 days	+	+	5α-reductase type I, WT PTEN	No report	University of Washington	[99]
RC-77T/E	Radical prostatectomy from an African-American patient	No report	+	+	p16, NKX3.1, β-catenin, α-actinin-1, filamin-A	8	Tuskegee University	[92]
Castration resistant								
PC-3	lumbar vertebral metastasis	33 h	−	−	PTEN loss, no p53 expression, TGF-α, EGFR, transferrin receptor	7, 8, 18, 19	ATCC, SIGMA, ACCEGEN, Creative Bioarray	[100]
DU-145	Brain metastasis	34 h	−	−	TGF-α/β, EGFR, IGF-1, EGF	5, 7, 8, 18	ATCC, ACCEGEN	[101]
C4-2/C4-2B	mouse vertebral metastasis LNCaP cell xenograft	48 h	+	+	p53, PTEN loss, marker chromosome m1	8	ATCC	[102,103]
22Rv1	CWR22R xenograft derivative	35–40 h	+	+	kallikrien-like serine protease, AR splice variant	8, 18	ATCC, SIGMA, ACCEGEN, Creative Bioarray	[104]
ARCaP	ascites fluid of a patient with advanced metastatic disease	No report	+	+	EGFR, c-erb B2/neu, c-erb B3, bombesin, serotonin	8, 18	Novicure Biotechnology	[105]

(* = Discontinued).

**Table 2:** The advantages and limitations of patient-derived xenograft models.

Model	Advantages	Limitations	Sources
3D-organoid	In vivo-like complexity Retain 3D architecture Maintain heterogeneity Good for high-throughput screening Good for drug response testing	Low establishment rate with primary hormone-sensitive tumor Success in only aggressive PCa specimens Lack vasculature Deficient microenvironment and immunity	Primary prostate cancer patient-derived tissue
PDX	Maintain heterogeneity Retain 3D architecture Intact endocrine system Includes microenvironment	Time-consuming and expensive Established in a mouse with deficient immunity Microenvironment is different from a human	Primary prostate cancer patient-derived tissue, CrownBio, The Jackson Laboratory
